# Pathogenesis of nonfamilial somatotroph adenomas

**DOI:** 10.1210/clinem/dgag116

**Published:** 2026-03-13

**Authors:** Anat Ben-Shlomo, Shlomo Melmed

**Affiliations:** Department of Medicine, Pituitary Center, Cedars-Sinai Health Sciences University, Los Angeles, CA 90048, USA; Multidisciplinary Adrenal Program, Department of Medicine, Cedars-Sinai Health Sciences University, Los Angeles, CA 90048, USA; Department of Medicine, Pituitary Center, Cedars-Sinai Health Sciences University, Los Angeles, CA 90048, USA

**Keywords:** pituitary adenoma, growth hormone, cAMP, GNAS, DNA damage

## Abstract

**Context:**

Excess growth hormone (GH) production leading to acromegaly most commonly emanates from an adenomatous pituitary somatotroph. Understanding the pathogenesis of these adenomas will elucidate how biologic behavior affects acromegaly treatment outcomes.

**Evidence acquisition:**

We searched PubMed for relevant English-language original research and review articles on signaling pathways and molecular drivers implicated in the pathogenesis of nonfamilial somatotroph adenomas in patients with acromegaly.

**Evidence synthesis:**

Somatotroph cells express cognate G protein–coupled receptors for both hypothalamic stimulatory GH-releasing hormone (GHRH) and inhibitory somatostatin. Somatotroph GH transcription and secretion, as well as somatotroph cell lineage development, proliferation, and differentiation, are mediated by GHRH signaling through its cognate receptor (growth hormone–releasing hormone receptor [GHRHR]), driving increased intracellular cyclic adenosine monophosphate (cAMP) levels. Point mutations in *GNAS* and other genomic and nongenomic aberrations in the tightly regulated GHRH–GHRHR signaling pathway result in persistent cAMP signaling, inducing GH production and somatotroph proliferation, and potentially favoring the development of sporadic somatotroph adenomas. Enhanced cAMP signaling also increases DNA damage markers and activates DNA damage response pathways, leading to a senescent adenomatous phenotype tightly linked to GH overproduction.

**Conclusion:**

The cAMP pathway appears to be a dominant molecular driver of somatotroph adenoma pathogenesis. Elevated cAMP drives GH hypersecretion and somatotroph proliferation and also induces DNA damage, as evidenced by increased genomic instability and a senescent signature. Collectively, these findings elucidate a molecular framework for the biological behavior of these adenomas and their responsiveness to therapies targeting cAMP-dependent pathways, including somatostatin receptor ligands.

Pituitary adenomas arise from highly differentiated anterior pituitary cell types, each engendering a unique clinical phenotype (1, 2). Pituitary somatotroph growth hormone (GH) synthesis occurs as a result of cell-specific transcription of the human *GH* gene regulated by neuroendocrine, intrapituitary, and peripheral stimulatory and inhibitory signals. Excess GH production leading to acromegaly most commonly emanates from an adenomatous pituitary somatotroph source, or rarely, from an extrapituitary neoplasm (3). Somatotroph adenomas are characterized by expression of GH and GH-specific transcription factors, as well as imaging morphology (2, 4, 5).

Somatotroph adenomas arise from monomorphous somatotroph cells expressing the specific *GH* gene transcription factor pituitary-specific positive transcription factor 1 (PIT1, also known as POU1F1), and may exhibit either dense or sparse cytoplasmic GH granules (Fig. 1**)**. Mixed bimorphous GH- and prolactin (PRL)–expressing adenomas, as well as monomorphous mixed somatolactotroph adenomas, coproduce GH and PRL. Less prevalent pituitary cell subtypes may also give rise to GH overproduction (2). Extrapituitary acromegaly is very rarely encountered and may be caused by ectopic tumor GH-releasing hormone (GHRH) production leading to somatotroph hyperplasia, eutopic hypothalamic tumor GHRH production, or ectopic GH production (1, 6). Iatrogenic acromegaly may also be encountered in patients inappropriately abusing GH.

**Figure 1:**
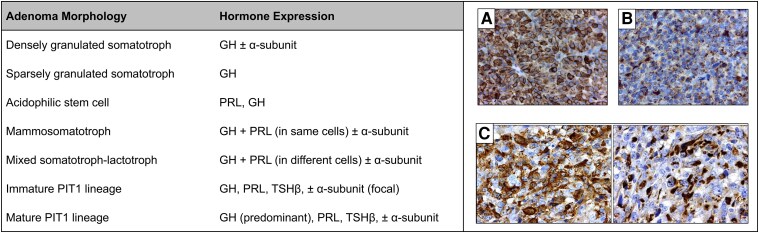
GH-secreting adenomas derived from the PIT1/POU1F1 lineage. Hormone expression detected on immunohistochemistry differs based on somatotroph adenoma morphology. Staining for GH is more prominent in (A) densely granulated somatotroph adenomas than in (B) sparsely granulated somatotroph adenoma. (C) Mixed somatotroph–lactotroph adenoma stains for GH (left) and PRL (right) in different cells. GH, growth hormone; PIT1, pituitary-specific positive transcription factor 1; PRL, prolactin; TSH, thyroid-stimulating hormone. Table adapted by permission of the Endocrine Society from Melmed et al (2); images (A) and (B) courtesy of Luis Syro, MD; image (C) reprinted with permission from *Medscape*, https://emedicine.medscape.com/article/1868045-overview, accessed January 7, 2026.

Sporadic somatotroph adenomas account for 95% of all somatotroph adenomas and are defined as occurring without an identifiable germline mutation in known predisposition genes and with no apparent family history of pituitary adenomas or associated syndromic features (7). Familial somatotroph adenomas, by contrast, arise in the setting of germline mutations (*AIP, MEN1, PRKAR1A, CDKN1B, GPR101,* and *SDHx*) or as part of recognized syndromes such as multiple endocrine neoplasia 1 (MEN1), Carney complex, McCune–Albright syndrome, or familial isolated pituitary adenoma (FIPA) (8-10).

Unfortunately, distinction between sporadic and familial adenomas in the literature is often unclear. First, the estimate that 5% of adenomas are familial predates our understanding of how predisposition genes contribute to adenoma pathogenesis. For example, germline *AIP* mutations are found in ∼4% of patients with adenomas initially classified as sporadic, and in up to 11% to 20% of young adults with macroadenomas or gigantism. Thus, a proportion of patients with adenomas labeled sporadic in earlier studies may, in fact, harbor undetected germline mutations (1, 11). Moreover, the frequency of novel genetic drivers associated with somatotroph adenomas in the general population remains undetermined (1, 10), and the incomplete penetrance of some mutations in FIPA kindreds can lead to underestimation of a genetic driver, especially when relying on patient-reported family history that may miss affected relatives who were undiagnosed or had subclinical disease (1, 12). Importantly McCune–Albright syndrome results from postzygotic (mosaic) *GNAS* mutations that are neither strictly germline nor somatic, creating a classification gray zone (8, 9).

Finally, selection of patients for genetic testing can vary among published studies, with some testing all patients and others testing only those meeting specific criteria, such as younger age, macroadenoma, or family history, leading to inconsistent classification across publications (1, 11). Until comprehensive genetic testing becomes standard practice, clear distinctions between sporadic and familial somatotroph tumors in the literature remains a challenge.

Regardless of acromegaly etiology, the biochemical disorder is characterized by high levels of both GH and resultant-induced insulin-like growth factor I (IGF-I), which dependently and independently expose peripheral tissues to adverse distal signaling, leading to a plethora of clinical acromegaly features and comorbidities. The structure–function relationships of somatotroph adenomas have enabled a functional classification of acromegaly subtypes determined by cluster analysis (3, 13). Type 1 comprises patients aged >60 years with small microadenomas, densely packed GH granules, mildly elevated GH and/or IGF-I levels, and a more indolent, less aggressive clinical phenotype. These patients usually exhibit favorable disease-specific mortality outcomes. By contrast, patients with Type 3 disease are younger, with sparsely granulated and larger, often invasive macroadenomas, higher hormone levels, more florid clinical comorbidities, and an adverse disease-specific mortality outcome. Patients with Type 2 disease show an intermediate phenotype.

Here, we critically review the current evidence favoring a hypothesis for the pathogenesis of nonfamilial somatotroph adenomas primarily driven by abnormal cyclic adenosine monophosphate (cAMP) signaling leading to both GH overproduction and benign somatotroph proliferation.

Whether or not pituitary neoplasms should be termed adenomas or neuroendocrine tumors has been debated (14, 15). Given the invariably benign nature of these lesions, as well as the need to prevent patient anxiety and their concern of inappropriately being classified as harboring a malignancy, we refer to GH-secreting adenomas of pituitary cell origin as somatotroph adenomas (2).

## Search strategy

We searched PubMed for relevant English-language original research and review articles published between January 1, 1980, and December 1, 2025, using the search terms “acromegaly,” “somatotroph,” “adenoma,” “pathophysiology,” “cyclic AMP,” “G protein subunit,” “GNAS,” “AIP,” “somatostatin,” “glucose-dependent insulinotropic polypeptide,” “ghrelin,” “phosphodiesterase,” “somatic copy number alterations,” and “DNA damage,” We selected mainly articles published between January 1, 2000, and December 31, 2025, as well as relevant and highly referenced earlier publications. We also searched the reference lists of articles identified by this search strategy and selected those we judged relevant. Our reference list was further modified based on comments from peer reviewers.

## Signaling pathways driving somatotroph function

**Figure 2:**
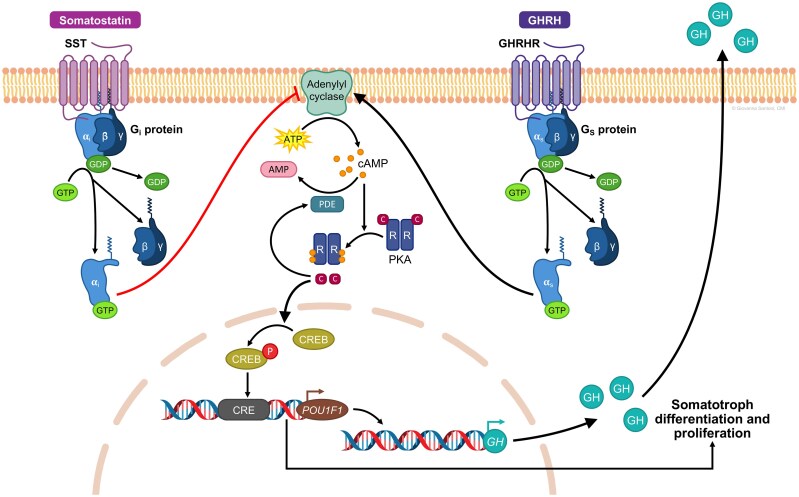
Regulation of somatotroph GH production. Cartoon depiction of intracellular signaling pathways mediating GH secretion and somatotroph differentiation and proliferation. α_i_, G protein subunit α_i_; β, G protein subunit β; γ, G protein subunit γ; AMP, adenosine monophosphate; ATP, adenosine triphosphate; C, catalytic subunit; cAMP, cyclic adenosine monophosphate; CRE, cAMP-response element; CREB, cAMP-response element binding protein; GDP, guanosine 5′-diphosphate; GH, growth hormone; GHRH, growth hormone–releasing hormone; GHRHR, growth hormone–releasing hormone receptor; GTP, guanosine 5′-triphosphate; P, phosphorylation; PIT1, pituitary-specific positive transcription factor 1; PKA, protein kinase A; R, regulatory subunit; SST, somatostatin receptor type. Adapted from Vélez EJ, Unniappan S. *Front Endocrinol (Lausanne)*. 2021;11:614981, © 2021 Vélez and Unniappan, under a Creative Commons CC BY license.

Despite a large body of published reports seeking common oncogene mutations leading to somatotroph adenoma initiation and growth, very rarely identified classic oncogenic driver mutations have not been validated as being functionally or uniformly relevant (9). Somatotrophs express cognate G protein–coupled receptors for both hypothalamic stimulatory GHRH and inhibitory somatostatin (16). Somatotroph GH transcription and secretion as well as somatotroph cell lineage development, proliferation, and differentiation are mediated by GHRH signaling through its cognate receptor (growth hormone–releasing hormone receptor [GHRHR]), driving increased intracellular cAMP levels (17-22) (Fig. 2). Pathway mutations, alterations in imprinting and receptor expression, and chaperone dysfunction lead to persistent cAMP signaling and altered calcium dynamics. In turn, these effects drive GH production and somatotroph proliferation, underpinning the pathogenesis of sporadic pituitary somatotroph adenomas.

Growth hormone–releasing hormone receptor, expressed predominantly in somatotroph membranes, initiates signaling through its associated G protein by promoting exchange of guanosine diphosphate for guanosine triphosphate (GTP) on the stimulatory G protein subunit α_s_ (Gα_s_). Activation of adenylyl cyclase leads to increased intracellular cAMP to regulate rapid cellular responses and long-term gene expression. cAMP binds to the regulatory subunits of the protein kinase A (PKA) holoenzyme, releasing catalytic subunits that phosphorylate diverse targets, leading to GH secretion and somatotroph cell proliferation and growth. Activated PKA stimulates sodium channels, depolarizing the somatotroph membrane and leading to voltage-dependent calcium influx, GH vesicle and cell membrane fusion, and GH release from secretory granules into the systemic circulation. Activated PKA also activates the cAMP-response element binding protein (CREB) at Ser^133^, which recruits transcriptional coactivators p300 and CREB-binding protein for target gene transcription, including for GH and GHRHR (23). PIT1, the somatotroph lineage-determining factor, contains cAMP promoter response elements, and CREB-Ser^133^ binding increases PIT1 transcription. Thus, activated PKA enhances GH production, as well as somatotroph lineage proliferation and differentiation (23, 24). Concurrently, the exchange protein activated by cAMP (EPAC) is induced to modulate cell adhesion, cytoskeletal organization, and mitogen-activated protein kinase (MAPK)/extracellularly regulated kinase signaling to fine-tune GH secretion and somatotroph proliferation (20, 21).

Spatial and temporal specificity of these signals is tightly controlled by phosphodiesterases (PDEs), especially the dominant PDE4 isoforms that degrade cAMP (25-27). Chaperones regulate assembly, localization, and stability of cAMP signaling machinery that generates, propagates, and terminates cAMP-induced responses. The cochaperone aryl hydrocarbon receptor–interacting protein (AIP), considered a tumor suppressor protein, interacts with PDE4 isoforms to constrain cAMP levels. Aryl hydrocarbon receptor–interacting protein thus maintains spatially restricted, tightly regulated cAMP signaling microdomains, ensuring fidelity of hormonal responses (28).

Somatostatin functions as the primary hypothalamic inhibitor of GH secretion, counterbalancing GHRH stimulatory effects to generate characteristic pulsatile GH secretion patterns. Somatostatin pulses are released from periventricular hypothalamic neurons, with high tone suppressing GH release during interpulse intervals and lower tone permitting GHRH-driven GH surges, particularly during sleep onset (19, 29). Somatotrophs express mainly somatostatin receptor type 2 (SST2) and SST5, which couple to G protein subunits α_i/o_ (Gα_i/o_) and exert rapid suppression of GH secretion. Activation of these receptors suppresses adenylyl cyclase, lowering intracellular cAMP and PKA, thereby antagonizing GHRH-induced signaling. In parallel, somatostatin opens potassium channels and inhibits voltage-gated calcium channels, leading to membrane hyperpolarization and reduced calcium influx required for exocytosis, while also disrupting exocytosis through the activation of phosphotyrosine phosphatases. These coordinated actions rapidly and reversibly suppress GH release without altering somatotroph differentiation (30).

## Molecular drivers of somatotroph adenoma pathogenesis

**Figure 3:**
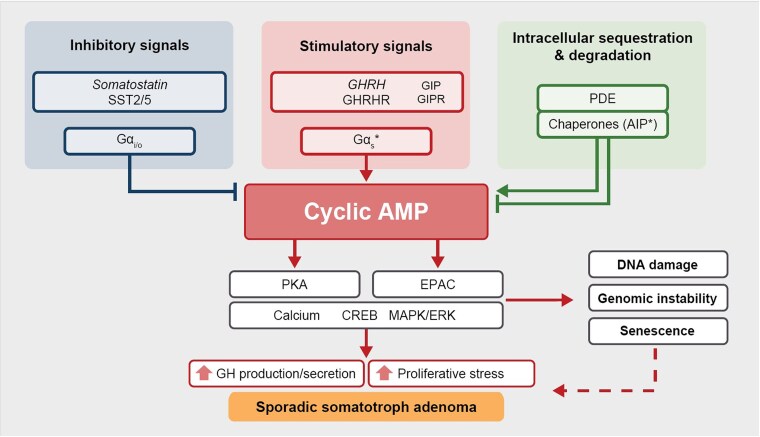
Drivers of nonfamilial somatotroph adenoma pathogenesis. Cartoon depiction of intracellular signaling pathways mediating somatotroph adenoma pathogenesis. Asterisks denote genes with established mutations known to lead to sporadic somatotroph adenoma pathogenesis. Dashed line denotes linked but not necessarily causal mechanism. AIP, aryl hydrocarbon receptor–interacting protein; AMP, adenosine monophosphate; CREB, cyclic adenosine monophosphate–response element binding protein; EPAC, exchange protein activated by cAMP; ERK, extracellularly regulated kinase; Gα_i_, G protein subunit α_i_; Gα_o_, G protein subunit α_o_; Gα_s_, G protein subunit α_s_; GH, growth hormone; GHRH, growth hormone–releasing hormone; GHRHR, growth hormone–releasing hormone receptor; GIP, glucose-dependent insulinotropic polypeptide; GIPR, glucose-dependent insulinotropic polypeptide receptor; MAPK, mitogen-activated protein kinase; PDE, phosphodiesterase; PKA, protein kinase A; SST, somatostatin receptor type.

Genomic and nongenomic aberrations in the tightly regulated GHRH–GHRHR signaling pathway result in persistent cAMP and calcium signaling, inducing GH production and somatotroph proliferation, and potentially favoring development of sporadic somatotroph adenomas (Fig. 3).

### Gα_S_ (GNAS)

#### GNAS mutations

In a subset of somatotroph adenomas, a mutated stimulatory Gα_s_ constitutively induces GH secretion and intracellular cAMP (31). Point mutations at codons Arg201 and Gln227 in somatotroph adenomas abolish intrinsic GTPase activity of Gα_s_, locking the protein in an active GTP-bound state and leading to constitutive stimulation of adenylyl cyclase and persistent cAMP elevation. Thus, the constitutively active “gsp oncogene” links dysregulated Gα_s_ signaling to excessive GH secretion and pituitary adenoma development (32, 33). The most common sporadic somatic pathogenic *GNAS* mutation hot spots, including R201C (Arg→Cys), R201H (Arg→His), R201S (Arg→Ser), Q227L (Gln→Leu), and Q227R (Gln→Arg) (34), are critical for GTP hydrolysis.

About 40% of sporadic somatotroph adenomas in the United States harbor a *GNAS* mutation (34). In 24 patients, a mutation at R201C was most common (62.5%) and Q227L was very rare (34). Worldwide, reported mutation rates vary widely, ranging from a low of 4.4% reported in Japan (35) to as high as 59.5% in Korea (36), underscoring the challenges in comparing rates across studies and the value of establishing standards for both testing methodology and patient selection. Strikingly, *GNAS* mutations are largely restricted to somatotroph adenomas and rarely found in other pituitary adenoma subtypes (37, 38), highlighting the centrality of cAMP signaling in the pathogenesis of sporadic somatotroph adenomas.

Several features distinguish *GNAS* mutation–positive from *GNAS* mutation–negative somatotroph adenomas. *GNAS* mutation–positive somatotroph adenomas occur more frequently in males, are often smaller and less invasive, and have higher preoperative IGF-I levels (33, 34, 39-42). Results of observational and retrospective studies indicate that *GNAS* mutation–positive somatotroph adenomas do not consistently demonstrate better long-term responses to somatostatin receptor ligands (SRLs). A meta-analysis found *GNAS* mutations were associated with greater GH reduction during acute octreotide testing (43), but a retrospective analysis (44) and a systematic review (45) found no association between *GNAS* mutation status and long-term biochemical control with SRL therapy.

The presence of *GNAS* mutations correlates inversely with cavernous sinus invasion and Ki-67 (37), and a meta-analysis found a greater reduction in GH during octreotide suppression testing (43). Patients with *GNAS* mutation–positive adenomas also showed more favorable GH lowering and remission rates after surgery (36).

#### GNAS imprinting

Although *GNAS* mutations are, to date, the sole rigorously identified driver of somatotroph adenoma development, upregulation of wild-type *GNAS* expression also has been implicated in its pathophysiology. Uniquely, the pituitary *GNAS* gene is imprinted (ie, expression derives predominantly from 1 allele [maternal], while the second [paternal] allele is generally silenced). Relaxation of the imprinted *GNAS* is more specific to sporadic somatotroph adenomas, regardless of whether they harbor *GNAS* mutations (46). Adenomas containing high levels of wild-type GNAS mRNA and protein were smaller and more responsive to SRL, supporting the role of relaxed *GNAS* imprinting in determining phenotype (47). Thus, aberrant imprinting may reflect an alternative route to GNAS overactivity, contributing to cAMP signaling dysregulation even in the absence of such mutations (48, 49).

### Glucose-dependent insulinotropic polypeptide

Glucose-dependent insulinotropic polypeptide (GIP), an incretin secreted by the proximal small intestine in response to nutrient ingestion, signals through the GIP receptor (GIPR) to activate Gα_s_ and adenylate cyclase, generate cAMP, and activate PKA and EPAC signaling (50). Two patients with somatotroph adenoma showed paradoxical GH increase after an oral glucose tolerance test (OGTT), and GH increases after GIP stimulation or a meal (51). Subsequently, in a cohort of 43 patients, 7 showed paradoxical GH increases during an OGTT, 3 of whom overexpressed GIPR mRNA and protein, independent of *GNAS* mutations; paradoxical responses disappeared postoperatively, suggesting adenoma-driven mechanisms. Transfection of GH-producing GH3 cells with human GIPR showed that GIP stimulation dose-dependently activated cAMP signaling and GH promoter activity, even in the absence of GIP, perhaps suggesting additional constitutive receptor activity (52).

Analysis of 25 somatotroph adenomas revealed high GIPR mRNA and protein levels in 40%, with both membrane and cytoplasmic staining colocalizing with GH. A paradoxical GH increase during an OGTT was evident in 80% of patients with high GIPR expression, and 60% of somatotroph adenomas with high GIPR expression showed in vitro GH secretion induced by GIP, correlating with GIPR levels. *GNAS* mutations, seen in 20% of adenomas, occurred exclusively in those expressing low levels of GIPR, suggesting mutual exclusivity between GIPR overexpression and *GNAS* mutations (53). The GIPR locus (19q13.32) showed no somatic mutations, flanking gene alterations, or somatic copy number alterations (SCNAs). Thus, GIPR overexpression is a distinct, *GNAS* mutation–independent mechanism driving cAMP activation and paradoxical GH secretion in acromegaly. A self-sustaining GH/IGF-I/GIP axis has been proposed to perpetuate cAMP signaling in GIPR-overexpressing somatotroph adenomas (53).

Infusion of the GIPR antagonist GIP(3-30)NH_2_ abolished a paradoxical GH increase in 4 of 7 patients with acromegaly, indicating that endogenous GIP may mediate paradoxical GH secretion. Mean age– and sex-adjusted IGF-I levels were higher in patients with paradoxical GH secretion vs those without, but circulating GIP levels did not differ between groups, suggesting receptor expression rather than ligand availability as the key determinant. Of 5 patients with paradoxical GH secretion, 3 who responded to GIPR antagonist expressed high GIPR protein levels whereas 2 expressed low levels; by contrast, 5 of 16 patients without the paradoxical GH secretion expressed GIPR abundantly. Glucose-dependent insulinotropic polypeptide stimulated cAMP and GH secretion in human GIPR-transfected GH3 cells, and GIP increased GH secretion in 16% of primary adenoma cultures (54).

These studies demonstrate that GIP drives paradoxical GH responses to an OGTT in a subset of patients with somatotroph adenomas, a finding associated with higher GIPR expression. Whether GIPR–cAMP pathway activation also contributes to somatotroph adenoma growth and clinical development remains unclear (55). A key unresolved question is whether GIPR overexpression is merely a functional secretory phenotype driving GH release or whether it also engages growth and survival programs that could influence tumor behavior, such as via sustained cAMP compartmentalization, EPAC-dependent signaling, or cross talk with MAPK/PI3K pathways. These and other questions are important and warrant further investigations.

### Ghrelin

Ghrelin, a stimulator of somatotroph GH secretion, acts through both hypothalamic and pituitary mechanisms (56). Ghrelin binds the constitutively active somatotroph GH secretagogue receptor 1a (GHS-R1a). This, in turn, activates Gα_q_/11-coupled phospholipase C, which increases inositol trisphosphate and intracellular calcium, leading to rapid exocytosis of GH-containing granules. Ghrelin acts synergistically with GHRH to enhance activation of the Gα_s_–cAMP–PKA pathway (57). Stimulation of hypothalamic GHRH neurons by ghrelin inhibits somatostatinergic tone, thereby amplifying stimulatory input to the pituitary (58).

Ghrelin and GHS-R1a transcripts are expressed in normal human pituitary and hypothalamic tissue; GHS-R1a mRNA is consistently and strongly expressed in somatotroph adenomas. However, its functional role in normal physiology and tumorigenesis remains unclear (59).

Treatment of somatotroph adenoma primary cultures with a GHS-R1a inverse agonist demonstrated dose-dependent decreased GH, an effect potentiated when combined with SRL. The agonist also reduced GH in cultured somatotroph adenomas, including some that showed octreotide resistance (60).

Evidence supporting a tumorigenic role for ghrelin remains limited and is derived largely from functional studies in primary cell cultures. Recurrent genomic alterations in GHS-R1a have not been consistently reported, suggesting that altered expression and/or signaling, rather than structural gene alteration, may underlie observed phenotypes.

### Phosphodiesterase

Phosphodiesterases regulate intracellular cAMP signaling by hydrolyzing it to its inactive form, 5'-adenosine monophosphate; tightly control cAMP pathway activation; and determine cAMP amplitude, duration, and compartmentalization (61). In somatotroph adenomas, expression of the PDE family is frequently increased, likely representing a compensatory response to chronically elevated cAMP signaling (62). In resected adenoma tissue, PDE4A and PDE4B expression was variable and unrelated to *GNAS* mutation status, whereas PDE4C and PDE4D transcripts were significantly higher in *GNAS* mutation–positive somatotroph adenomas. PDE8B mRNA, undetectable in normal pituitary autopsy specimens, was expressed in nearly all somatotroph adenomas and at significantly higher levels in *GNAS* mutation–positive adenomas. Short-term PDE inhibition markedly increased CREB phosphorylation in *GNAS* mutation–positive somatotroph adenomas, suggesting that elevated PDE activity functionally restrains cAMP/PKA–CREB signaling in these adenomas (62-65).

### AIP

The *AIP* gene, located on chromosome 11q13.3, is a tumor susceptibility gene mutation detected in about 15% of FIPA kindreds (11). *AIP* mutations are very rare in sporadic somatotroph adenomas (66, 67), identified in <4% (68) to 13% (69) when young patients with macroadenomas are selected, and therefore may be more important in apparently sporadic young-onset adenomas. Beyond a chaperone role, AIP selectively interacts with PDE4A5 and PDE2A, linking cyclic nucleotide signaling to aryl hydrocarbon receptor regulation (70, 71). Aryl hydrocarbon receptor–interacting protein binds PDE4A5 at R271, increases PDE4A5 sensitivity, and attenuates PKA-mediated phosphorylation of PDE4A5 at Ser^140^. Thus, AIP acts as a selective and multifunctional regulator of PDE4A5, likely for fine-tuning cAMP signaling. *AIP* mutations such as R271W impair this interaction, implicating AIP in the control of proliferative cAMP pathways relevant to somatotroph tumorigenesis (70). However, precise downstream tumorigenic pathways remain incompletely defined (11, 64).

## The role of GHRH and somatostatin in somatotroph adenoma growth

### GHRH and GHRHR

Unlike normal somatotrophs, adenomatous somatotrophs show disrupted GH secretory patterns, with abnormalities in pulsatility, amplitude, and frequency. Following adenoma resection and biochemical remission, GH secretory dynamics may revert to the healthy physiological pattern (72, 73). Residual alterations, particularly reduced pulse amplitude, irregular periodicity, and increased basal secretory fraction, suggest incomplete restoration of hypothalamic control and long-lasting changes in the hypothalamic somatostatin–GHRH regulatory axis in some patients (72).

To date, germline or somatic *GHRHR* gene mutations have not been implicated in the pathogenesis of somatotroph adenomas (74-77). Chronic excess GHRH production, such as that seen in patients with overproduction of GHRH from ectopic (ie, bronchial or pancreatic neuroendocrine tumors) or hypothalamic (ie, gangliocytomas) tumors, leads to pituitary somatotroph hyperplasia and rarely to adenomatous transformation (78-81). This trophic effect has been recapitulated in mice overexpressing the human *GHRH* transgene, which develop somatotroph hyperplasia progressing to adenomas (82). Thus, chronic excess exposure of the somatotroph cell to GHRH action is a potent driver of somatotroph hyperplasia and may contribute to adenoma formation.

### Somatostatin and its cognate Receptors

Mutations in the somatostatin (SST) gene or in SST receptor genes *SST1*–*5* are not established drivers of sporadic somatotroph adenomas (74-76, 83). A single report showed a somatotroph adenoma with an *SST5* missense mutation (R240W) linked to octreotide resistance (84). More recently, a 121-tumor proteogenomic cohort reported nonrecurrent *SST5* mutations in 3 patients and copy number gains of *SST5*, but without hotspot patterns or causal proof (77), and *SST5* mutations did not correlate with GH suppression during the octreotide loading test or with adenoma shrinkage following preoperative SRL therapy. Thus, while SST5 expression varies between somatotroph adenoma subtypes, *SST5* gene mutations are likely incidental, and unlikely to be functionally significant drivers of adenoma phenotype (77).

*SST5* single-nucleotide polymorphisms, notably P335L, have been associated with susceptibility to somatotroph adenomas, tumor behavior, and reduced responsiveness to SRL, suggesting pharmacogenetic rather than tumorigenic roles (85). Similarly, a truncated *SST5* splice variant (sst5TMD4) behaves as a functional antagonist and correlates with poor SRL response in pituitary adenomas (86). Accordingly, contemporary reviews of somatotroph adenomas emphasize SST2/SST5 receptor expression, epigenetic regulation, and downstream signaling as determinants of phenotype and drug response, with no reproducible role for mutations in *SST* or its receptors in tumor initiation (87, 88).

## Genomic variations, DNA damage, and senescence

Although classic oncogenic point mutation drivers have not been reported for somatotroph adenomas, they consistently exhibit a low point mutation burden and a high prevalence of chromosomal aberrations as key molecular features (89). Several studies have demonstrated widespread SCNAs in somatotroph adenomas, as compared with low SCNA frequency in nonfunctioning gonadotroph adenomas (37, 74-77, 89-94). Notably, however, the thresholds used to define “low,” “moderate,” and “high” SCNA burden are not standardized across studies. Therefore, it remains difficult to determine whether SCNA burden and genomic instability promote a distinct tumorigenic pathway or reflect a shared mechanistic axis with cAMP activation (Fig. 3).

Whole-genome sequencing and single-nucleotide variant array analyses on 12 somatotroph adenomas demonstrated very few coding single-nucleotide variants (mean 2.3 per tumor) and larger structural variants; *GNAS* p.Arg201Cys was the only recurrent mutation. These results highlighted somatic alterations in calcium and adenosine triphosphate signaling pathways rather than novel recurrent driver genes. Recurrent whole-chromosome losses (including chromosomes 1, 6, 13, 14, 15, 16, 18, and 22) were common, supporting a pathogenic role for chromosomal copy number alterations and possibly epigenetic mechanisms, rather than high mutational burden (74).

Further supporting these findings, an analysis of 20 somatotroph adenomas showed a very low somatic mutation burden, while *GNAS* mutation was confirmed as the main recurrent driver. In a subset of these adenomas, a recurrent mutation in GRB10, an adaptor protein involved in growth factor and insulin signaling, also was identified. Somatic copy number alterations manifesting as recurrent chromosomal gains and losses were more common than point mutations (37).

Importantly, there are several reports of a lower SCNA burden in *GNAS* mutation–positive somatotroph adenomas. Using whole exome sequencing, canonical cancer-associated somatic mutations profiling, and fluorescence in situ hybridization (FISH), 42 somatotroph adenomas were found to be frequently “genomically disrupted,” characterized by extensive arm-level SCNAs. Frequent losses of chromosomes 1p and 11 occurred, and ∼40% of disrupted adenomas showed evidence of whole-genome doubling. Transcriptomic analysis revealed that disrupted adenomas exhibited increased variability and enrichment of chromosomal instability gene signatures, linking large-scale genomic alterations to altered transcriptional programs (75). In a follow-up analysis using next-generation sequencing, 65% of 300 genes analyzed in 114 adenomas were mutated, and genes in the DNA damage pathway were most frequently altered. Of note, 59 adenomas (52%) were null cell, 21 (18%) were GH-secreting, and 14 (12%) each secreted PRL and adrenocorticotrophic hormone, and mutation counts differed based on functional status and adenoma subtype. *GNAS* mutations were identified in 6 of 21 somatotroph adenomas and gain of chromosome 20q was common in *GNAS* mutation–negative adenomas (90).

In another study of 43 somatotroph adenomas stratified by SCNA burden, all *GNAS* mutation–positive somatotroph adenomas belonged to the low-SCNA group, whereas no adenoma *GNAS* mutations were detected in the high-SCNA group. In the latter group, catastrophic chromothripsis was found in a single adenoma with a germline *AIP* mutation (chromosome 10q) and in an adenoma from a patient with gigantism (chromosomes 2q, 11p, 11q, and 15q). These chromothripsis events involved tens of SCNAs and numerous breakpoints, affecting oncogenes (eg, *TLX1*, *FGFR2*, and *TCF7L2*), tumor suppressors (eg, *SUFU* and *VENTX*), and genes involved in chromosome segregation and DNA crosslink repair (eg, *BARD1*, *SMC3*, and *XRCC5*). DNA FISH confirmed extensive intratumoral SCNA heterogeneity, with multiple cell subpopulations differing in chromosomal copy number states (91).

A multiomic study of 23 somatotroph adenomas showed a moderate degree of chromosomal alterations (mean, 18% of the genome altered), at higher rates compared with silent adenomas, and mainly characterized by arm-level rather than focal events. Epigenetically, PIT1/POU1F1 lineage-somatotroph adenomas showed global DNA hypomethylation, particularly in “open sea” regions >4 kb from any CpG island, as well as high expression of the MEG3 microRNA cluster. Methylation and chromosomal alterations were negatively correlated, and molecular heterogeneity was reported between densely and sparsely granulated somatotroph adenomas (92).

In 56 somatotrophs out of 195 pituitary adenomas analyzed, *GNAS* mutations 11p.Arg201Cys and 2p.Gln227Leu were identified in 25% of patients, while an additional 13 *GNAS* mutation–negative somatotroph adenomas showed gain of the 20q13.32 region, encompassing the *GNAS* locus; these gains were validated by FISH in all tested cases. *GNAS* mutations and chromosome 20q gains were not determined by patient sex, age, adenoma grade, secretion status, or size, suggesting distinct but clinically similar subgroups. Large arm-level or whole-chromosome gains were common but not associated with *GNAS* mutation status. Silent somatotroph adenomas had no detectable SCNA, whereas actively secreting somatotroph adenomas showed significantly more alterations, linking genomic instability to hormonal activity. Neither the quantity nor pattern of SCNA predicted recurrence, but somatotroph adenomas lacking *GNAS* mutation or chromosome 20q gain tended to recur more frequently (93).

Using targeted capture sequencing and copy number analysis of 36 genes and proteogenomics of 121 somatotroph adenomas, *GNAS* mutations were identified as a driver, detected in 57% of tumors, primarily at classical hotspots (p.Arg201 and p.Gln227). Additional low-frequency mutations were found in *AIP*, *GPR101*, *SST5*, *ARRB1/2*, *PRKACA*, *PRKACB*, and *PRKAR1A*, along with copy number gains in *SST5* and *GPR101* and losses in *PRKACB* and *SDHx* genes. Clinically, *GNAS* mutation–positive somatotroph adenomas were smaller, less invasive, and more responsive to SRL and dopamine agonists, consistent with higher DRD2 expression, yet postoperative outcomes were similar (77).

We analyzed 159 pituitary adenomas using whole exome sequencing; in accordance with other studies, we detected characteristic widespread SCNAs despite a low overall single-nucleotide mutation burden (76). Somatotroph adenomas (n & 35) exhibited the highest SCNA ratios among pituitary adenoma types, typically involving large chromosomal amplifications or deletions. *GNAS* mutations were present in 29% of somatotroph adenomas that carried low- to medium-degree SCNAs, with none showing high-degree SCNAs. *GNAS* mutation–negative somatotroph adenomas often showed medium or high SCNA levels. Pathway analyses of single-gene SCNA revealed that the cAMP signaling pathway was most frequently affected in hormone-secreting adenomas, accounting for 55% of all cAMP pathway single-gene SCNAs identified across the cohort. This pathway did not reach significance in somatotroph adenomas, likely due to sample size. In somatotroph adenomas, the Fanconi anemia DNA repair pathway was significantly enriched for gene deletions (eg, *BRCA1*, *BRCA2*, *REV3L*, *HES1*, and *RMI1*).

Mechanistic experiments using primary mouse pituitary cultures demonstrated that elevated cAMP signaling induced by forskolin or the GHRH analog CJC-129 rapidly increased DNA damage markers, functionally linking cAMP pathway activation to genomic instability (76). This induced DNA damage was partially attenuated by octreotide, consistent with its cAMP inhibitory effects. Confirming earlier observations (95), somatotroph adenomas showed elevated p53 and p21^Wif1/Cip1^ mRNA and protein levels compared with gonadotroph/null cell adenomas, indicating activation of DNA damage response pathways (76).

Functionally, somatotroph adenomas display increased senescence activity, with β-galactosidase abundance correlating with GH expression, and treatment of primary tumor cultures with nutlin, a p53 activator, further enhancing GH expression and secretion alongside p53 and p21^Wif1/Cip1^ induction (96). Pituitary tumor-transforming gene, the index mammalian securin (97), is abundant in somatotroph adenomas, leading to non-faithful chromosome separation, and is associated with induced p53- and p21^Wif1/Cip1^-mediated senescence. These findings may underlie the mechanism for strongly constrained somatotroph malignant transformation (95). Thus, somatotroph cellular senescence may act as a restraining buffer, explaining the invariably benign nature of somatotroph adenomas with the exceedingly rare progression to true malignancy. Collectively, these findings link sustained cAMP pathway activation with genomic instability and DNA damage responses, leading to a senescent adenomatous phenotype tightly linked to GH overproduction.

In summary, these reports provide supporting evidence for somatotroph adenomas as being genetically “quiet” at the point mutation level but genomically “loud” at the structural level, possibly with *GNAS* mutations defining a major molecular class, and chromosomal instability defining another. Whether DNA damage activation and the senescent phenotype are the cause or the result of cAMP activation–induced somatotroph adenomatous transformation is yet to be determined. Furthermore, given the putative role of GHRHR-mediated signaling in the pathogenesis of the somatotroph adenoma, it remains unclear why small GH-secreting adenomas are surrounded by nonhyperplastic somatotroph cells (3). As complete resection of these microadenomas may, in fact, result in complete resolution of acromegaly signs and symptoms, with high remission rates, the evidence strongly favors an inherent somatotroph cell defect rather than a hypothalamic defect underlying pathogenesis of the disorder.

## Conclusions

Sustained activation of the cAMP pathway appears to be a dominant molecular driver of somatotroph adenoma pathogenesis. Specifically, activating *GNAS* mutations at Arg201 or Gln227, which lock Gα_s_ in its active state, produce constitutive adenylyl cyclase stimulation and elevated cAMP, while aberrant *GNAS* imprinting can upregulate wild-type *GNAS* expression in the absence of coding mutations. Other mechanisms include ectopic expression of *GIPR*, which activates cAMP signaling in response to GIP and induces paradoxical GH secretion; synergistic ghrelin–GHRH signaling through GHS-R1a; and impaired cAMP regulation due to *PDE* alterations or *AIP* loss-of-function. Furthermore, elevated cAMP drives GH hypersecretion and somatotroph proliferation and also induces replication stress and DNA damage, as evidenced by increased genomic instability, SCNAs, Fanconi pathway genes, p53 and p21^Wif1/Cip1^ activation, and a senescent signature. Collectively, these findings describe a molecular framework for the biological behavior of these adenomas and their responsiveness to therapies targeting cAMP-dependent pathways, including SRL.

## Data Availability

Data sharing is not applicable to this article as no data sets were generated or analyzed during the present study.

## References

[1] Melmed S . Pituitary-tumor endocrinopathies. N Engl J Med. 2020;382(10):937‐950. 32130815 10.1056/NEJMra1810772

[2] Melmed S, Kaiser UB, Lopes MB, et al Clinical biology of the pituitary adenoma. Endocr Rev. 2022;43(6):1003‐1037. 35395078 10.1210/endrev/bnac010 PMC9695123

[3] Melmed S, Braunstein GD, Horvath E, Ezrin C, Kovacs K. Pathophysiology of acromegaly. Endocr Rev. 1983;4(3):271‐290. 6354702 10.1210/edrv-4-3-271

[4] Colao A, Grasso LFS, Giustina A, et al Acromegaly. Nat Rev Dis Primers. 2019;5(1):20. 30899019 10.1038/s41572-019-0071-6

[5] Ho KKY, Fleseriu M, Wass J, et al A proposed clinical classification for pituitary neoplasms to guide therapy and prognosis. Lancet Diabetes Endocrinol. 2024;12(3):209‐214. 38301678 10.1016/S2213-8587(23)00382-0 PMC12051483

[6] Melmed S . Acromegaly pathogenesis and treatment. J Clin Invest. 2009;119(11):3189‐3202. 19884662 10.1172/JCI39375 PMC2769196

[7] Marrero-Rodriguez D, Moscona-Nissan A, Sidauy-Adissi J, et al The molecular biology of sporadic acromegaly. Best Pract Res Clin Endocrinol Metab. 2024;38(3):101895. 38641464 10.1016/j.beem.2024.101895

[8] Boguslawska A, Korbonits M. Genetics of acromegaly and gigantism. J Clin Med. 2021;10(7):1377. 33805450 10.3390/jcm10071377 PMC8036715

[9] De Sousa SMC, Lenders NF, Lamb LS, Inder WJ, McCormack A. Pituitary tumours: molecular and genetic aspects. J Endocrinol. 2023;257(3):e220291. 36951812 10.1530/JOE-22-0291

[10] Giustina A, Colao A. Acromegaly. N Engl J Med. 2025;393(19):1926‐1939. 41223366 10.1056/NEJMra2409076

[11] Beckers A, Daly AF. The clinical, pathological, and genetic features of familial isolated pituitary adenomas. Eur J Endocrinol. 2007;157(4):371‐382. 17893250 10.1530/EJE-07-0348

[12] Fleseriu M, Langlois F, Lim DST, Varlamov EV, Melmed S. Acromegaly: pathogenesis, diagnosis, and management. Lancet Diabetes Endocrinol. 2022;10(11):804‐826. 36209758 10.1016/S2213-8587(22)00244-3

[13] Cuevas-Ramos D, Carmichael JD, Cooper O, et al A structural and functional acromegaly classification. J Clin Endocrinol Metab. 2015;100(1):122‐131. 25250634 10.1210/jc.2014-2468 PMC4283008

[14] Ho KKY, Kaiser UB, Chanson P, et al Pituitary adenoma or neuroendocrine tumour: the need for an integrated prognostic classification. Nat Rev Endocrinol. 2023;19(11):671‐678. 37592077 10.1038/s41574-023-00883-8 PMC12519436

[15] Rindi G, Klimstra DS, Abedi-Ardekani B, et al A common classification framework for neuroendocrine neoplasms: an International Agency for Research on Cancer (IARC) and World Health Organization (WHO) expert consensus proposal. Mod Pathol. 2018;31(12):1770‐1786. 30140036 10.1038/s41379-018-0110-y PMC6265262

[16] Lechan RM . Neuroendocrinology. In: Melmed S, Auchus RJ, Goldfine AB, Rosen CJ, Kopp PA, eds. Williams Textbook of Endocrinology. 15^th^ ed. Elsevier; 2024:93‐166.

[17] Mayo KE, Miller T, DeAlmeida V, Godfrey P, Zheng J, Cunha SR. Regulation of the pituitary somatotroph cell by GHRH and its receptor. Recent Prog Horm Res. 2000;55:266‐237. discussion 266-237. 11036940

[18] Halmos G, Szabo Z, Dobos N, Juhasz E, Schally AV. Growth hormone-releasing hormone receptor (GHRH-R) and its signaling. Rev Endocr Metab Disord. 2025;26(3):343‐352. 39934495 10.1007/s11154-025-09952-x PMC12137518

[19] Steyn FJ, Tolle V, Chen C, Epelbaum J. Neuroendocrine regulation of growth hormone secretion. Compr Physiol. 2016;6(2):687‐735. 27065166 10.1002/cphy.c150002

[20] Pertuit M, Barlier A, Enjalbert A, Gerard C. Signalling pathway alterations in pituitary adenomas: involvement of Gsalpha, cAMP and mitogen-activated protein kinases. J Neuroendocrinol. 2009;21(11):869‐877. 19732293 10.1111/j.1365-2826.2009.01910.x

[21] Peverelli E, Mantovani G, Lania AG, Spada A. cAMP in the pituitary: an old messenger for multiple signals. J Mol Endocrinol. 2013;52(1):R67‐R77. 24049068 10.1530/JME-13-0172

[22] Formosa R, Vassallo J. cAMP signalling in the normal and tumorigenic pituitary gland. Mol Cell Endocrinol. 2014;392(1-2):37‐50. 24845420 10.1016/j.mce.2014.05.004

[23] Mayr B, Montminy M. Transcriptional regulation by the phosphorylation-dependent factor CREB. Nat Rev Mol Cell Biol. 2001;2(8):599‐609. 11483993 10.1038/35085068

[24] Bertherat J . Nuclear effects of the cAMP pathway activation in somatotrophs. Horm Res. 1997;47(4-6):245‐250. 9167959 10.1159/000185471

[25] Smith FD, Langeberg LK, Scott JD. The where's and when's of kinase anchoring. Trends Biochem Sci. 2006;31(6):316‐323. 16690317 10.1016/j.tibs.2006.04.009

[26] Conti M, Beavo J. Biochemistry and physiology of cyclic nucleotide phosphodiesterases: essential components in cyclic nucleotide signaling. Annu Rev Biochem. 2007;76:481‐511. 17376027 10.1146/annurev.biochem.76.060305.150444

[27] Baillie GS . Compartmentalized signalling: spatial regulation of cAMP by the action of compartmentalized phosphodiesterases. FEBS J. 2009;276(7):1790‐1799. 19243430 10.1111/j.1742-4658.2009.06926.x

[28] Schernthaner-Reiter MH, Trivellin G, Stratakis CA. Chaperones, somatotroph tumors and the cyclic AMP (cAMP)-dependent protein kinase (PKA) pathway. Mol Cell Endocrinol. 2020;499:110607. 31586652 10.1016/j.mce.2019.110607

[29] Montero-Hidalgo AJ, Rio-Moreno D, Perez-Gomez M, Luque JM, Kineman RM, D R. Update on regulation of GHRH and its actions on GH secretion in health and disease. Rev Endocr Metab Disord. 2025;26(3):305‐320. 39838154 10.1007/s11154-025-09943-y

[30] Ben-Shlomo A, Melmed S. Pituitary somatostatin receptor signaling. Trends Endocrinol Metab. 2010;21(3):123‐133. 20149677 10.1016/j.tem.2009.12.003 PMC2834886

[31] Vallar L, Spada A, Giannattasio G. Altered Gs and adenylate cyclase activity in human GH-secreting pituitary adenomas. Nature. 1987;330(6148):566‐568. 2825031 10.1038/330566a0

[32] Landis CA, Masters SB, Spada A, Pace AM, Bourne HR, Vallar L. GTPase inhibiting mutations activate the alpha chain of Gs and stimulate adenylyl cyclase in human pituitary tumours. Nature. 1989;340(6236):692‐696. 2549426 10.1038/340692a0

[33] Buchfelder M, Fahlbusch R, Merz T, Symowski H, Adams EF. Clinical correlates in acromegalic patients with pituitary tumors expressing GSP oncogenes. Pituitary. 1999;1(3-4):181‐185. 11081196 10.1023/a:1009905131334

[34] Freda PU, Chung WK, Matsuoka N, et al Analysis of GNAS mutations in 60 growth hormone secreting pituitary tumors: correlation with clinical and pathological characteristics and surgical outcome based on highly sensitive GH and IGF-I criteria for remission. Pituitary. 2007;10(3):275‐282. 17594522 10.1007/s11102-007-0058-2

[35] Hosoi E, Yokogoshi Y, Hosoi E, et al Analysis of the Gs alpha gene in growth hormone-secreting pituitary adenomas by the polymerase chain reaction-direct sequencing method using paraffin-embedded tissues. Acta Endocrinol (Copenh). 1993;129(4):301‐306. 8237246 10.1530/acta.0.1290301

[36] Jung H, Kim K, Kim D, et al Associations of GNAS mutations with surgical outcomes in patients with growth hormone-secreting pituitary adenoma. Endocrinol Metab (Seoul). 2021;36(2):342‐350. 33752302 10.3803/EnM.2020.875 PMC8090461

[37] Song ZJ, Reitman ZJ, Ma ZY, et al The genome-wide mutational landscape of pituitary adenomas. Cell Res. 2016;26(11):1255‐1259. 27670697 10.1038/cr.2016.114 PMC5099864

[38] Tatsi C, Stratakis CA. The genetics of pituitary adenomas. J Clin Med. 2019;9(1):30. 31877737 10.3390/jcm9010030 PMC7019860

[39] Barlier A, Gunz G, Zamora AJ, et al Pronostic and therapeutic consequences of Gs alpha mutations in somatotroph adenomas. J Clin Endocrinol Metab. 1998;83(5):1604‐1610. 9589663 10.1210/jcem.83.5.4797

[40] Kim HJ, Kim MS, Park YJ, et al Prevalence of Gs alpha mutations in Korean patients with pituitary adenomas. J Endocrinol. 2001;168(2):221‐226. 11182759 10.1677/joe.0.1680221

[41] Larkin S, Reddy R, Karavitaki N, Cudlip S, Wass J, Ansorge O. Granulation pattern, but not GSP or GHR mutation, is associated with clinical characteristics in somatostatin-naive patients with somatotroph adenomas. Eur J Endocrinol. 2013;168(4):491‐499. 23288882 10.1530/EJE-12-0864

[42] Yang Y, Yao Y, Deng K, et al Somatic GNAS mutations in acromegaly: prevalence, clinical features and gender differences. Endocr Connect. 2025;14(1):e240266. 39513543 10.1530/EC-24-0266 PMC11728932

[43] Efstathiadou ZA, Bargiota A, Chrisoulidou A, et al Impact of gsp mutations in somatotroph pituitary adenomas on growth hormone response to somatostatin analogs: a meta-analysis. Pituitary. 2015;18(6):861‐867. 26115707 10.1007/s11102-015-0662-5

[44] Wildemberg LE, Henriques D, Elias PCL, et al Gsp mutation is not a molecular biomarker of long-term response to first-generation somatostatin receptor ligands in acromegaly. Cancers (Basel). 2021;13(19):4857. 34638340 10.3390/cancers13194857 PMC8508484

[45] Dillon BR, Ruddy M, McQuade EC, et al Clinical characteristics associated with somatic GNAS mutations in acromegaly: a systematic review and institutional experience. Front Endocrinol (Lausanne). 2026;17:1736208. 41648725 10.3389/fendo.2026.1736208 PMC12867796

[46] Hayward BE, Barlier A, Korbonits M, et al Imprinting of the G(s)alpha gene GNAS1 in the pathogenesis of acromegaly. J Clin Invest. 2001;107(6):R31‐R36. 11254676 10.1172/JCI11887 PMC208949

[47] Picard C, Silvy M, Gerard C, et al Gs alpha overexpression and loss of Gs alpha imprinting in human somatotroph adenomas: association with tumor size and response to pharmacologic treatment. Int J Cancer. 2007;121(6):1245‐1252. 17514647 10.1002/ijc.22816

[48] Weinstein LS, Yu S, Warner DR, Liu J. Endocrine manifestations of stimulatory G protein alpha-subunit mutations and the role of genomic imprinting. Endocr Rev. 2001;22(5):675‐705. 11588148 10.1210/edrv.22.5.0439

[49] Mantovani G, Lania AG, Spada A. GNAS imprinting and pituitary tumors. Mol Cell Endocrinol. 2010;326(1-2):15‐18. 20398730 10.1016/j.mce.2010.04.009

[50] Muller TD, Adriaenssens A, Ahren B, et al Glucose-dependent insulinotropic polypeptide (GIP). Mol Metab. 2025;95:102118. 40024571 10.1016/j.molmet.2025.102118 PMC11931254

[51] Umahara M, Okada S, Ohshima K, Mori M. Glucose-dependent insulinotropic polypeptide induced growth hormone secretion in acromegaly. Endocr J. 2003;50(5):643‐650. 14614222 10.1507/endocrj.50.643

[52] Occhi G, Losa M, Albiger N, et al The glucose-dependent insulinotropic polypeptide receptor is overexpressed amongst GNAS1 mutation-negative somatotropinomas and drives growth hormone (GH)-promoter activity in GH3 cells. J Neuroendocrinol. 2011;23(7):641‐649. 21554434 10.1111/j.1365-2826.2011.02155.x

[53] Regazzo D, Losa M, Albiger NM, et al The GIP/GIPR axis is functionally linked to GH-secretion increase in a significant proportion of gsp(-) somatotropinomas. Eur J Endocrinol. 2017;176(5):543‐553. 28179449 10.1530/EJE-16-0831

[54] Jensen MH, Gasbjerg LS, Skov-Jeppesen K, et al GIP receptor antagonism eliminates paradoxical growth hormone secretion in some patients with acromegaly. J Clin Endocrinol Metab. 2025;110(3):715‐729. 39172542 10.1210/clinem/dgae583 PMC11834721

[55] Dalle Nogare M, Avallone S, Galletta E, et al GIPR in GH-PitNETs: molecular and functional insights. Endocr Relat Cancer. 2025;32(10):250106. 10.1530/ERC-25-0106 41066443

[56] van der Lely AJ, Tschop M, Heiman ML, Ghigo E. Biological, physiological, pathophysiological, and pharmacological aspects of ghrelin. Endocr Rev. 2004;25(3):426‐457. 15180951 10.1210/er.2002-0029

[57] Cunha SR, Mayo KE. Ghrelin and growth hormone (GH) secretagogues potentiate GH-releasing hormone (GHRH)-induced cyclic adenosine 3',5'-monophosphate production in cells expressing transfected GHRH and GH secretagogue receptors. Endocrinology. 2002;143(12):4570‐4582. 12446584 10.1210/en.2002-220670

[58] Tschop M, Smiley DL, Heiman ML. Ghrelin induces adiposity in rodents. Nature. 2000;407(6806):908‐913. 11057670 10.1038/35038090

[59] Korbonits M, Bustin SA, Kojima M, et al The expression of the growth hormone secretagogue receptor ligand ghrelin in normal and abnormal human pituitary and other neuroendocrine tumors. J Clin Endocrinol Metab. 2001;86(2):881‐887. 11158061 10.1210/jcem.86.2.7190

[60] Mear Y, Blanchard MP, Defilles C, et al Ghrelin receptor (GHS-R1a) and its constitutive activity in somatotroph adenomas: a new co-targeting therapy using GHS-R1a inverse agonists and somatostatin analogs. J Clin Endocrinol Metab. 2014;99(12):E2463‐E2471. 25272306 10.1210/jc.2014-2753

[61] Kelly MP, Nikolaev VO, Gobejishvili L, et al Cyclic nucleotide phosphodiesterases as drug targets. Pharmacol Rev. 2025;77(3):100042. 40081105 10.1016/j.pharmr.2025.100042 PMC13095499

[62] Bizzi MF, Bolger GB, Korbonits M, Ribeiro-Oliveira A. Phosphodiesterases and cAMP pathway in pituitary diseases. Front Endocrinol (Lausanne). 2019;10:141. 30941100 10.3389/fendo.2019.00141 PMC6433792

[63] Lania A, Persani L, Ballare E, Mantovani S, Losa M, Spada A. Constitutively active Gs alpha is associated with an increased phosphodiesterase activity in human growth hormone-secreting adenomas. J Clin Endocrinol Metab. 1998;83(5):1624‐1628. 9589667 10.1210/jcem.83.5.4814

[64] Bizzi MF, Pinheiro SVB, Bolger GB, et al Reduced protein expression of the phosphodiesterases PDE4A4 and PDE4A8 in AIP mutation positive somatotroph adenomas. Mol Cell Endocrinol. 2018;476:103‐109. 29729370 10.1016/j.mce.2018.04.014

[65] Persani L, Borgato S, Lania A, et al Relevant cAMP-specific phosphodiesterase isoforms in human pituitary: effect of Gs(alpha) mutations. J Clin Endocrinol Metab. 2001;86(8):3795‐3800. 11502813 10.1210/jcem.86.8.7779

[66] Yu R, Bonert V, Saporta I, Raffel LJ, Melmed S. Aryl hydrocarbon receptor interacting protein variants in sporadic pituitary adenomas. J Clin Endocrinol Metab. 2006;91(12):5126‐5129. 17018653 10.1210/jc.2006-1731

[67] Leontiou CA, Gueorguiev M, van der Spuy J, et al The role of the aryl hydrocarbon receptor-interacting protein gene in familial and sporadic pituitary adenomas. J Clin Endocrinol Metab. 2008;93(6):2390‐2401. 18381572 10.1210/jc.2007-2611

[68] Cazabat L, Bouligand J, Salenave S, et al Germline AIP mutations in apparently sporadic pituitary adenomas: prevalence in a prospective single-center cohort of 443 patients. J Clin Endocrinol Metab. 2012;97(4):E663‐E670. 22319033 10.1210/jc.2011-2291

[69] Tichomirowa MA, Barlier A, Daly AF, et al High prevalence of AIP gene mutations following focused screening in young patients with sporadic pituitary macroadenomas. Eur J Endocrinol. 2011;165(4):509‐515. 21753072 10.1530/EJE-11-0304

[70] Bolger GB, Peden AH, Steele MR, et al Attenuation of the activity of the cAMP-specific phosphodiesterase PDE4A5 by interaction with the immunophilin XAP2. J Biol Chem. 2003;278(35):33351‐33363. 12810716 10.1074/jbc.M303269200

[71] de Oliveira SK, Hoffmeister M, Gambaryan S, Muller-Esterl W, Guimaraes JA, Smolenski AP. Phosphodiesterase 2A forms a complex with the co-chaperone XAP2 and regulates nuclear translocation of the aryl hydrocarbon receptor. J Biol Chem. 2007;282(18):13656‐13663. 17329248 10.1074/jbc.M610942200

[72] Bajuk Studen K, Barkan A. Assessment of the magnitude of growth hormone hypersecretion in active acromegaly: reliability of different sampling models. J Clin Endocrinol Metab. 2008;93(2):491‐496. 18029464 10.1210/jc.2007-1451 PMC2243233

[73] van der Lely AJ, Harris AG, Lamberts SW. The sensitivity of growth hormone secretion to medical treatment in acromegalic patients: influence of age and sex. Clin Endocrinol (Oxf). 1992;37(2):181‐185. 1395069 10.1111/j.1365-2265.1992.tb02304.x

[74] Valimaki N, Demir H, Pitkanen E, et al Whole-genome sequencing of growth hormone (GH)-secreting pituitary adenomas. J Clin Endocrinol Metab. 2015;100(10):3918‐3927. 26280510 10.1210/jc.2015-3129

[75] Bi WL, Horowitz P, Greenwald NF, et al Landscape of genomic alterations in pituitary adenomas. Clin Cancer Res. 2017;23(7):1841‐1851. 27707790 10.1158/1078-0432.CCR-16-0790 PMC5380512

[76] Ben-Shlomo A, Deng N, Ding E, et al DNA damage and growth hormone hypersecretion in pituitary somatotroph adenomas. J Clin Invest. 2020;130(11):5738‐5755. 32673291 10.1172/JCI138540 PMC7598090

[77] Yamato A, Nagano H, Gao Y, et al Proteogenomic landscape and clinical characterization of GH-producing pituitary adenomas/somatotroph pituitary neuroendocrine tumors. Commun Biol. 2022;5(1):1304. 36435867 10.1038/s42003-022-04272-1 PMC9701206

[78] Garby L, Caron P, Claustrat F, et al Clinical characteristics and outcome of acromegaly induced by ectopic secretion of growth hormone-releasing hormone (GHRH): a French nationwide series of 21 cases. J Clin Endocrinol Metab. 2012;97(6):2093‐2104. 22442262 10.1210/jc.2011-2930

[79] Zendran I, Gut G, Kaluzny M, Zawadzka K, Bolanowski M. Acromegaly caused by ectopic growth hormone releasing hormone secretion: a review. Front Endocrinol (Lausanne). 2022;13:867965. 35757397 10.3389/fendo.2022.867965 PMC9218487

[80] Borson-Chazot F, Garby L, Raverot G, Claustrat F, Raverot V, Sassolas G. Acromegaly induced by ectopic secretion of GHRH: a review 30 years after GHRH discovery. Ann Endocrinol (Paris). 2012;73(6):497‐502. 23122576 10.1016/j.ando.2012.09.004

[81] Fainstein-Day P, Ullmann TE, Dalurzo MCL, Sevlever GE, Smith DE. The clinical and biochemical spectrum of ectopic acromegaly. Best Pract Res Clin Endocrinol Metab. 2024;38(3):101877. 38413286 10.1016/j.beem.2024.101877

[82] Kineman RD, Teixeira LT, Amargo GV, Coschigano KT, Kopchick JJ, Frohman LA. The effect of GHRH on somatotrope hyperplasia and tumor formation in the presence and absence of GH signaling. Endocrinology. 2001;142(9):3764‐3773. 11517152 10.1210/endo.142.9.8382

[83] Gunther T, Tulipano G, Dournaud P, et al International union of basic and clinical pharmacology. CV. Somatostatin receptors: structure, function, ligands, and new nomenclature. Pharmacol Rev. 2018;70(4):763‐835. 30232095 10.1124/pr.117.015388 PMC6148080

[84] Ballare E, Persani L, Lania AG, et al Mutation of somatostatin receptor type 5 in an acromegalic patient resistant to somatostatin analog treatment. J Clin Endocrinol Metab. 2001;86(8):3809‐3814. 11502816 10.1210/jcem.86.8.7787

[85] Ciganoka D, Balcere I, Kapa I, et al Identification of somatostatin receptor type 5 gene polymorphisms associated with acromegaly. Eur J Endocrinol. 2011;165(4):517‐525. 21810856 10.1530/EJE-11-0416 PMC3178914

[86] Duran-Prado M, Saveanu A, Luque RM, et al A potential inhibitory role for the new truncated variant of somatostatin receptor 5, sst5TMD4, in pituitary adenomas poorly responsive to somatostatin analogs. J Clin Endocrinol Metab. 2010;95(5):2497‐2502. 20233783 10.1210/jc.2009-2247

[87] Cuevas-Ramos D, Fleseriu M. Somatostatin receptor ligands and resistance to treatment in pituitary adenomas. J Mol Endocrinol. 2014;52(3):R223‐R240. 24647046 10.1530/JME-14-0011

[88] Botelho L, Dezonne RS, Wildemberg LE, Miranda RL, Gadelha MR, Andreiuolo F. Somatostatin receptors in pituitary somatotroph adenomas as predictors of response to somatostatin receptor ligands: a pathologist's perspective. Brain Pathol. 2025;35(1):e13313. 39473262 10.1111/bpa.13313 PMC11669419

[89] Srirangam Nadhamuni V, Korbonits M. Novel insights into pituitary tumorigenesis: genetic and epigenetic mechanisms. Endocr Rev. 2020;41(6):821‐846. 32201880 10.1210/endrev/bnaa006 PMC7441741

[90] Bi WL, Greenwald NF, Ramkissoon SH, et al Clinical identification of oncogenic drivers and copy-number alterations in pituitary tumors. Endocrinology. 2017;158(7):2284‐2291. 28486603 10.1210/en.2016-1967 PMC5505210

[91] Hage M, Viengchareun S, Brunet E, et al Genomic alterations and complex subclonal architecture in sporadic GH-secreting pituitary adenomas. J Clin Endocrinol Metab. 2018;103(5):1929‐1939. 29474559 10.1210/jc.2017-02287

[92] Neou M, Villa C, Armignacco R, et al Pangenomic classification of pituitary neuroendocrine tumors. Cancer Cell. 2020;37(1):123‐134. 31883967 10.1016/j.ccell.2019.11.002

[93] Lasolle H, Elsensohn MH, Wierinckx A, et al Chromosomal instability in the prediction of pituitary neuroendocrine tumors prognosis. Acta Neuropathol Commun. 2020;8(1):190. 33168091 10.1186/s40478-020-01067-5 PMC7653703

[94] Zhang F, Zhang Q, Zhu J, et al Integrated proteogenomic characterization across major histological types of pituitary neuroendocrine tumors. Cell Res. 2022;32(12):1047‐1067. 36307579 10.1038/s41422-022-00736-5 PMC9715725

[95] Chesnokova V, Zonis S, Kovacs K, et al P21(Cip1) restrains pituitary tumor growth. Proc Natl Acad Sci U S A. 2008;105(45):17498‐17503. 18981426 10.1073/pnas.0804810105 PMC2577704

[96] Chesnokova V, Zhou C, Ben-Shlomo A, et al Growth hormone is a cellular senescence target in pituitary and nonpituitary cells. Proc Natl Acad Sci U S A. 2013;110(35):E3331‐E3339. 23940366 10.1073/pnas.1310589110 PMC3761588

[97] Pei L, Melmed S. Isolation and characterization of a pituitary tumor-transforming gene (PTTG). Mol Endocrinol. 1997;11(4):433‐441. 9092795 10.1210/mend.11.4.9911

